# Text Mining Genotype-Phenotype Relationships from Biomedical Literature for Database Curation and Precision Medicine

**DOI:** 10.1371/journal.pcbi.1005017

**Published:** 2016-11-30

**Authors:** Ayush Singhal, Michael Simmons, Zhiyong Lu

**Affiliations:** National Center for Biotechnology Information (NCBI), National Library of Medicine (NLM), National Institutes of Health (NIH), Bethesda, Maryland, United States of America; University of Chicago, UNITED STATES

## Abstract

The practice of precision medicine will ultimately require databases of genes and mutations for healthcare providers to reference in order to understand the clinical implications of each patient’s genetic makeup. Although the highest quality databases require manual curation, text mining tools can facilitate the curation process, increasing accuracy, coverage, and productivity. However, to date there are no available text mining tools that offer high-accuracy performance for extracting such triplets from biomedical literature. In this paper we propose a high-performance machine learning approach to automate the extraction of disease-gene-variant triplets from biomedical literature. Our approach is unique because we identify the genes and protein products associated with each mutation from not just the local text content, but from a global context as well (from the Internet and from all literature in PubMed). Our approach also incorporates protein sequence validation and disease association using a novel text-mining-based machine learning approach. We extract disease-gene-variant triplets from all abstracts in PubMed related to a set of ten important diseases (breast cancer, prostate cancer, pancreatic cancer, lung cancer, acute myeloid leukemia, Alzheimer’s disease, hemochromatosis, age-related macular degeneration (AMD), diabetes mellitus, and cystic fibrosis). We then evaluate our approach in two ways: (1) a direct comparison with the state of the art using benchmark datasets; (2) a validation study comparing the results of our approach with entries in a popular human-curated database (UniProt) for each of the previously mentioned diseases. In the benchmark comparison, our full approach achieves a 28% improvement in F_1_-measure (from 0.62 to 0.79) over the state-of-the-art results. For the validation study with UniProt Knowledgebase (KB), we present a thorough analysis of the results and errors. Across all diseases, our approach returned 272 triplets (disease-gene-variant) that overlapped with entries in UniProt and 5,384 triplets without overlap in UniProt. Analysis of the overlapping triplets and of a stratified sample of the non-overlapping triplets revealed accuracies of 93% and 80% for the respective categories (cumulative accuracy, 77%). We conclude that our process represents an important and broadly applicable improvement to the state of the art for curation of disease-gene-variant relationships.

## Author Summary

To provide personalized health care it is important to understand patients’ genomic variations and the effect these variants have in protecting or predisposing patients to disease. Several projects aim at providing this information by manually curating such genotype-phenotype relationships in organized databases using data from clinical trials and biomedical literature. However, the exponentially increasing size of biomedical literature and the limited ability of manual curators to discover the genotype-phenotype relationships “hidden” in text has led to delays in keeping such databases updated with the current findings. The result is a bottleneck in leveraging valuable information that is currently available to develop personalized health care solutions. In the past, a few computational techniques have attempted to speed up the curation efforts by using text mining techniques to automatically mine genotype-phenotype information from biomedical literature. However, such computational approaches have not been able to achieve accuracy levels sufficient to make them appealing for practical use. In this work, we present a highly accurate machine-learning-based text mining approach for mining complete genotype-phenotype relationships from biomedical literature. We test the performance of this approach on ten well-known diseases and demonstrate the validity of our approach and its potential utility for practical purposes. We are currently working towards generating genotype-phenotype relationships for all PubMed data with the goal of developing an exhaustive database of all the known diseases in life science. We believe that this work will provide very important and needed support for implementation of personalized health care using genomic data.

## Introduction

Many genetic mutations protect or predispose individuals to disease [1]. The practice of precision medicine involves identifying such mutations in patients and modifying patient treatment to reflect each individual’s unique physiologic risks and strengths [2]. Databases of gene-disease relationships play a key role in this process by acting as a reference to which providers may refer to determine the significance of their patients’ mutations [3, 4]. In a similar way, these databases also play a key role in translational research [5, 6].

Currently, the highest quality databases require manual curation, often in conjunction with support from automated systems [7, 8]. Creating entries in these databases requires substantial human investment. For example, in the UniProt Knowledgebase (UniProtKB), each mutation receives a multitude of annotations providing information about gene function, role in disease, and molecular interactions, and other things [9]. These manual curation efforts are critical because the biomedical literature is a unique source of genotype-phenotype information. Curations in genotype-phenotype databases are also important components of synthesized knowledge bases of clinically actionable genetic information such as the Reference Variant Store (RVS) [6]. Yet, the high cost of expert curation of UniProtKB and databases alike is a rate-limiting factor for content coverage and updates.

Computational approaches to gene curation could potentially relieve the bottleneck of human resources in disease mutation annotation. Fully automating the curation system remains beyond the capacity of even state-of-the-art text mining systems, but automation of parts of the process is feasible. A separate, yet important role of text mining in database gene curation is that text mining tools can shed perspective on the scope and breadth of current databases by summarizing the entirety of relevant information in the biomedical literature.

Identification of genotype-phenotype relationships is a key concern in both clinical and research communities. Several well-known databases employ manual curation of biomedical literature to provide comprehensive coverage of such relationships in humans. Examples of these include OMIM [10], HGMD [11], Comparative Toxicogenomics Database (CTD) [12], GHR (http://ghr.nlm.nih.gov/) and UniProtKB [9]. Recent efforts in the direction of (semi-)automated approaches to facilitate database curation of genotype-phenotype relationships include extraction of sequence variation information from biomedical text. Overall, most methods confine their scope to mutation entity extraction without exploring the relationships of those mutations to other entities, such as diseases or genes. Examples of mutation recognition tools include MutationFinder [13], tmVar [14], and several others [15].

Several groups, however, have addressed variant relationship mining in text. One early, notable method developed by Kuipers et al [16] introduced an automatic method for extracting and validating mutations for a single disease–Fabry disease. Since their approach finds mutations on a single gene (GLA) at Xq22.1 for Fabry disease, it uses regular expression to identify mutation mentions and assumes them to be related to the disease and gene. Other early approaches to relationship extraction include, MuGeX [17], EnzyMiner [18], and OSIRIS [19]. Each of these has been reported with limitations and over-specialization [20]. More recently, Hakenberg et al developed an approach to mining a variety of pharmacogenomic relationships from PubMed abstracts [21]. Their work is particularly noteworthy in its comparison of text-mined results to a manually curated database–PharmGKB. Laurila et al mined functional impact information about gene variants [22], and Macintyre et al created a rule-based approach to identifying gene-disease and gene-variant relationships from literature for the purpose of investigating the impact of intergenic (non-coding) variants [23]. Of all the works on mutation relationship extraction, one of the most notable is the EMU tool developed by Doughty et al [20]. EMU provides a semi-automated approach to extract disease-related mutations from PubMed abstracts and full text. This work, which truly addresses broad genotype-phenotype relationship extraction is most comparable to our present work.

In all the above automated approaches, there are two common limitations: (a) disease-to-mutation relationships in the text are not explicitly detected or utilized for extraction; rather, a relationship is typically assumed (i.e. co-occurrence); (b) none of the above mentioned approaches explicitly focuses on extracting a three-way relationship between gene, mutation and the disease from the text. Regarding the latter limitation, the EMU tool can extract three-way relationships from text, but its primary focus is mutation extraction. Its gene and disease association extraction functionality is limited. Likewise, the works by Macintyre et al [23] and Hakenberg et al [21] include extraction of both gene-disease relations and gene-variant relations, but extraction of the entire triplet is not expressly evaluated.

**Fig 1:**
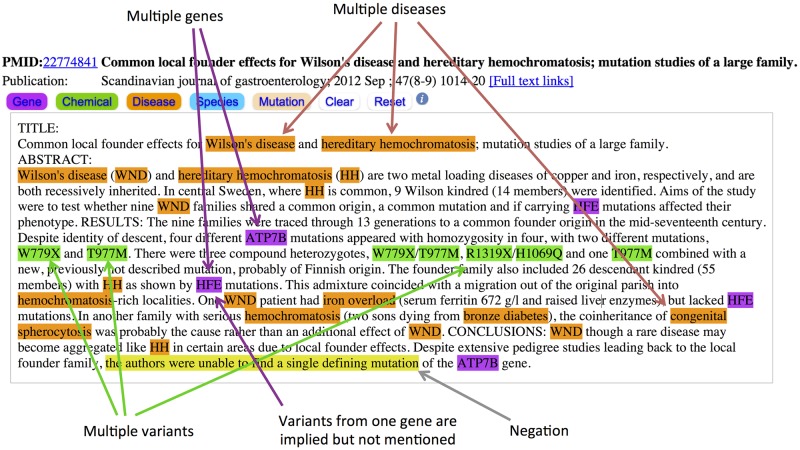
An example showing the complexity of mining triplet information from a PubMed abstract.

We have previously approached parts of this problem as well. One work [24] used a machine learning approach to determine disease-mutation association from PubMed abstracts. In another study [25] we experimented with crowd-based human judgment to determine binary associations between genes and mutations in PubMed abstracts. These previous works separately addressed identification of disease-mutation associations and gene-mutation associations, but neither attempted extraction of complete disease-gene-variant triplets. Developing an efficient, robust and fully automated approach to extract a full three-way relationship or triplet of disease-gene-variant from text is still challenging for several reasons. Firstly, correctly mining complex bio-entities from biomedical literature has been a long-standing challenge. Secondly, mining three-way relationships is even more complicated than mining two-way relationships. The challenges in gene-variant-disease extraction are heightened due to several factors concerning natural language processing and information presentation in biomedical literature. An example explaining such challenges is shown in Fig 1. As shown in the figure, a common challenge of text mining this information is that a single abstract may contain references to multiple diseases, genes, and mutations. The conventional, co-occurrence-based approach of assuming hereditary hemochromatosis (HH) to be related to all the mutations found in an abstract frequently results in false positives, as it does in this example. For this reason it is important to examine the text to establish gene-mutation and disease-mutation relationships. Fig 1 contains an abstract retrieved through a PubMed search for HH with various entities annotated using PubTator. In this abstract all named variants belong to the ATP7B gene and have a proposed association with Wilson disease (not HH). Researchers used known HH-causing variants in the HFE gene (none of which are mentioned in the abstract) to identify people in a population of Wilson disease patients who may also have had HH. A third disease–congenital spherocytosis–is cited as a confounding factor in their analysis. The author’s ultimate conclusion is that their methodologies were insufficient to definitively demonstrate a defining association between Wilson disease and variants of the gene in question.

In this paper we propose a novel, end-to-end approach to automate the extraction of disease-gene-variant triplets from biomedical literature. We employed our previously published supervised machine-learning approach to detect relationships between disease and mutation mentions in text. Then we mined gene association information using a novel approach that leverages global context (using PubMed and the Web via Bing search) followed by gene sequence validation in order to identify an exact gene match for the mutation. The end result of the proposed approach is a disease-gene-variant triplet. We describe the development of this system and analyze its performance in extracting gene mutations for a set of ten common diseases set forth in the list provided in Doughty et al [20] (breast cancer, prostate cancer, pancreatic cancer, lung cancer, acute myeloid leukemia, Alzheimer’s disease, hemochromatosis, age-related macular degeneration (AMD), diabetes mellitus, and cystic fibrosis). The performance of the proposed approach is evaluated in two ways: (1) direct comparison with the previous EMU approach using a benchmark dataset; (2) validation with a popular human-curated database (UniProtKB). The main contributions of this work are as follows:

1. Development of a novel framework for extracting full disease-gene-variant triplet information from text.
2. Proposal of a novel, global-context-based (as opposed to a local, co-occurrence-based) text mining approach to mine gene associations.
3. Testing the performance of our approach on ten common diseases using all relevant PubMed data for each disease.
4. Development of a new human-annotated corpus containing 430 disease-gene-variant triplets with their corresponding PMIDs. This corpus may be used by future researchers for building machine learning models as well as for performance evaluation.

## Methods

### Dataset used

The dataset used in this work is comprised of PubMed articles. Although the technique developed is applicable for any disease, we have analyzed and presented results for a set of ten diseases [20]. For each disease, we assembled a “Disease_corpus” by collecting a list of PMIDs from PubMed using the following query: “disease_name [tiab] AND English [26] AND has_abstract [filter]”. For each PMID in the Disease_corpus, we collected the PubMed title, abstract and annotation results for gene, mutation and disease mentions via PubTator [27]. In PubTator, the gene, mutation and disease annotations were extracted by GNormPlus [28], tmVar [14] and DNorm [29], respectively.

**Fig 2:**
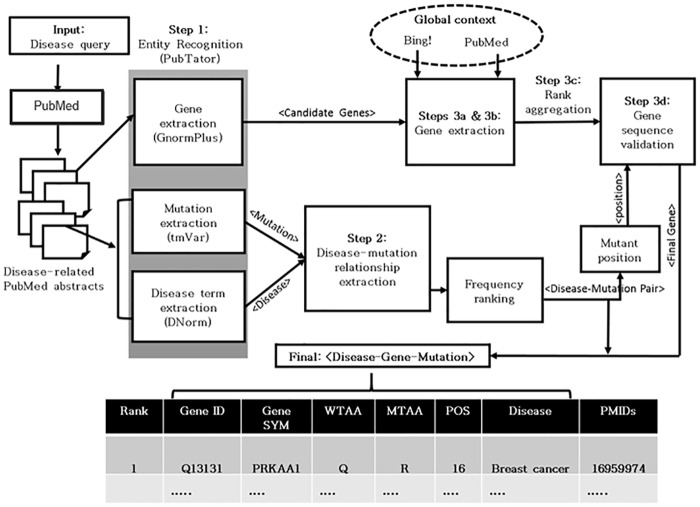
Overview of the proposed approach.

Our approach for identifying gene-disease-mutation relationships is portrayed schematically in Fig 2 and can be summarized as follows: Step 1: Identify all diseases, genes, and mutations in PubMed abstracts; Step 2: Associate mutations with a given disease from all articles in PubMed about that disease; and Step 3: Link the proteins or genes with each mutation using an aggregation of information extracted from PubMed, the Web (using Bing search engine features), and sequence analysis. This process results in a list of triplets of the form <disease, gene, variant>. Details of each step are as follows:

#### Step 1: Identifying disease, gene and mutation mentions

We used PubTator to identify disease, gene and mutation mentions. PubTator employs the following entity recognition tools for these tasks:

**GNormPlus**. GNormPlus extracts gene/proteins from a given text in two steps. In the first step it recognizes the gene/protein entity mention using a novel CRF++ [30] based module in combination with the species recognition module SR4GN [31]. In the second step, the gene/protein mention is normalized using GenNorm [32] in combination with the composite mention simplification tool, SimConcept [33] and an abbreviation resolution tool Ab3P [34].

**tmVar**. tmVar uses a conditional random field (CRF) model to detect mutation mentions in text [14]. The CRF model identifies the mutation type, reference amino acid or nucleotide, mutant nucleotide or amino acid and the mutation position as the states for the CRF i.e. each component of a mutation is treated as an individual state in the CRF and thus sequentially retrieved. The information about these components is learned from a curated tmVar corpus. Some additional rare mutation mentions are handled in the post-processing module.

**DNorm**. DNorm segments the given text into sentences and identifies disease mentions using BANNER [35]. The mentions are then normalized to disease concepts in MeSH by finding the best match using a learning-to-rank technique [29]. Finally the disambiguation related to primary disease or a disease synonym is resolved using a disease concept hierarchy. The output is a tuple of disease mention text span and corresponding concept (MeSH ID).

#### Step 2: Disease-Mutation relationship extraction

The details of this step can be found in our earlier work [24]; this information is also available in the supplementary material, S1 Text. In summary, mutations associated with the queried disease are identified using a machine-learning(ML)-based classification algorithm trained to detect disease-related mutations using a novel feature set derived by mining the text in PubMed abstracts. The feature set captures information such as mutation and disease mention proximity, disease mention frequency, same-sentence mention of mutation and disease and text sentiment (polarity) to distinguish between disease-related and non-related mutation mentions in text. The output of this step is a list of PMID-mutation pairs that are predicted to be related to the target disease. In our previous work, we tested our ML model and showed that retraining ML classifiers with disease-specific training sets is not necessary for classification of disease-mutation relationships in unrelated diseases, e.g. we trained a model using the prostate cancer training set and successfully applied it to finding relevant mutations for AMD. Therefore, for all diseases in this work (except breast cancer, which has its own training data available), we use the prostate cancer training set to build the ML model.

In the second step of the disease-mutation identification process, the PMID-mutation pairs for the disease are converted into an ordered list. Unique mutations are ranked in descending order by the number of PMIDs in which each mutation appears, and a corresponding list of referencing PMIDs is provided for each mutation. We refer to this list as the “Mutation-list”. This list features later in our gene-ranking process. The tmVar algorithm extracts all types of mutations, including insertions, deletions, and substitutions. The tmVar also recognizes multiple levels of mutation nomenclature, including DNA, RNA, and protein. However, for comparison purposes with UniProt KB (which curates only protein substitutions), we emphasize protein substitutions throughout the remaining text. Moreover, protein substitutions account for the majority of the total extracted mutations. We have provided information about all other types of mutations mined by our text mining approach in the supplementary material, S2 Text.

#### Step 3: Gene-variant relationship extraction

**Fig 3:**
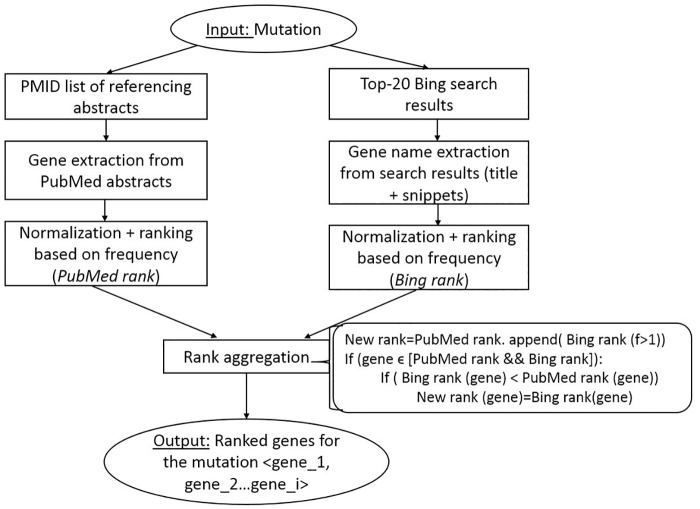
A schematic of identifying a ranked list of genes using global knowledge for a given mutation.

Once the disease-mutations are identified (Step 1), the next step is to extract the gene associated with each mutation. Performing this step within the local textual and structural information of individual abstracts is challenging and error-prone because (a) automatic gene recognition remains quite difficult and (b) in many cases abstracts mention multiple genes and mutations. Thus, we designed a three-step approach that takes advantage of global information beyond a single abstract. The steps for this approach are summarized in Fig 3, and the details are described below:

1. Gene extraction from a PubMed corpus (PubMed Rank): Given a mutation and a referencing PMID list, all genes (normalized to Entrez Gene ID) mentioned in the PubMed abstracts of corresponding PMIDs are collected and then ranked based on the aggregate score of their mentions in the local abstract as well as in all other abstracts. For example, if a gene is mentioned three times in one abstract, two times in another and one time in a third abstract, the aggregate score for that gene would be six. Scoring genes in this fashion allows all the candidate proteins for a given mutation to be ranked and listed in descending order by aggregate score. We call the resulting candidate list the *PubMed Rank List*. Using all related PMIDs to identify the correct gene match for the mutation, we minimize the effect of errors of our automatic gene tagger and increase the likelihood of finding a correct match since the correct gene may be mentioned repeatedly in multiple PMIDs.
2. Gene extraction from a Web corpus (Bing Rank): Parallel to the process described above, a *Bing Rank list* of candidate genes is also generated from the Web using the Bing search engine’s free API service [36]. The most frequent mention for each entry from the Mutation-list (obtained from the referencing abstracts) is given as the input query to the Bing search engine. Gene mentions for each query are mined from the title and text snippets of the top-20 Bing search results. We utilize the full text search feature provided by Bing without mining the full text because the relevant information is displayed by the search engine in the form of web-snippets. All gene mentions that occur in Bing text snippets containing mutation mentions are mined and added to the candidate list. Finally, the gene mentions extracted from the search snippets are normalized to Entrez Gene IDs and then ranked in descending order of frequency. We call the resulting candidate list the *Bing Rank List*. Since this list is generated using Web search results, it is based on global knowledge beyond the biomedical literature. We present an analysis of the effect of incorporating Bing into our ranking strategy in the supplementary material, S3 Text.
3. Rank aggregation: As shown in Fig 3, the two candidate lists of genes, *PubMed Rank List* and *Bing Rank List* are combined to develop a unique list of candidate genes for each disease-mutation pair. All genes with a frequency greater than one ((f>1) that occur only in the *Bing Rank List* are appended directly to the end of the *PubMed Rank List*. By doing so, the genes extracted from PubMed are assumed to be more relevant than those extracted from Bing. This is based on our observation that the Bing search results include noisier text, hence more false positive genes. The gene names that overlap between *Bing Rank List* and *PubMed Rank List* are therefore more important and their ranks need to be aggregated. For such genes, we simply raise their rank order in the *PubMed Rank List* by substituting the PubMed rank with the Bing Rank if they had a higher rank order in the *Bing Rank List* than in the *PubMed rank List*.
4. Sequence validation: in the final step of determining the associated gene for a given variant-disease pair, the exact match is identified from the candidate genes (from step 3.c) by sequence analysis of the gene, similar to the sequence filter step in Doughty et. al [20]. This analysis requires matching the reference or mutant amino acid (AA) or nucleotide in the protein or the gene sequence on the mutation position mentioned in the mutation. If the AA in the protein sequence at a mutation position (mut_pos) matches with the reference or mutant AA in the mutation, then that gene (gene_i) is marked as the exact match for the mutation. All protein isoforms of a gene were considered; a match in any protein isoform results in gene selection. Otherwise, the next gene in the candidate list is checked and so on until a match is found. In cases where none of the proteins provides an exact match, the topmost ranked protein is selected. We used the used NCBI’s gene2refseq to obtain the protein isoforms for genes. The protein sequence was obtained from FASTA data from NCBI’s RefSeq database [37].

## Results

In this section, we describe the experiments performed to assess the performance of the proposed approach. The analysis proceeds in two ways: (1) to assess the validity of our approach, we performed an intrinsic evaluation and comparison with the state of the art using a gold-standard benchmark dataset; (2) to assess the utility of our approach, we compared the results of our approach with entries in a popular human-curated database (UniProt) for ten diseases.

### Intrinsic evaluation: Comparison with EMU

The performance of the proposed approach is first evaluated for disease-gene-variant triplet extraction from biomedical literature on the benchmark datasets for prostate and breast cancer used to report the performance of the EMU approach [20]. These datasets consist of manually annotated lists of disease-gene-variant triplets from 203 and 141 PubMed articles for breast and prostate cancer respectively. Using these benchmark datasets, we report the accuracies of our approach in standard measures (precision, recall and F-measure) and compare the accuracy of our text-mined triplets (cross-validation based) directly with the results of EMU.

Table 1 displays a comparison between our proposed approach and the previous state of the art (EMU). The table includes results with and without sequence filters as well as results for a simple co-occurrence relationship extraction baseline that uses our entity tagging tools. Since EMU also used a co-occurrence approach but with different entity tagging tools, this co-occurrence column allows a comparison of the performance of the different entity tagging tools. As shown in Table 1, our entity tagging tools performed similarly to those of EMU. Without sequence filters, our approach achieves marked improvement over the results of EMU in precision and overall F_1_-measure on both datasets, although EMU has better recall. Employing a sequence filter improves the precision of EMU at the expense of substantial decreases in recall (The EMU with sequence filter removes gene-variant pairs that fail the protein sequence validation check, reducing the overall number of mutations extracted by EMU and therefore the recall also decreases), but this tradeoff does not occur in our approach. Rather, the use of a sequence filter improves all three metrics.

**Table 1:** Comparison of proposed approach with EMU approach on benchmark datasets. The parantheses values correspond to (true positives, false positives) for precision and (true positive, false negatives) for recall.

Corpus	EMU: Without sequence filter	Our baseline approach: Co-occurrence only	Our approach: Without sequence filter	EMU: With sequence filter	Our full approach: With sequence filter
**PCA** Precision	0.39 (151, 237)	0.37 (154, 263)	0.75 (132, 42)	0.59 (127, 89)	**0.82 (144, 32)**
Recall	0.80 (151, 37)	**0.82 (154, 34)**	0.70 (132, 56)	0.66 (127, 61)	0.77 (144, 44)
F-measure	0.52	0.51	0.724	0.62	**0.794**
**BCA** Precision	0.34 (242, 470)	0.33 (252, 504)	0.738 (206, 73)	0.61 (193, 121)	**0.742 (207, 72)**
Recall	0.85 (242, 42)	**0.89 (252, 32)**	0.725 (206, 78)	0.68 (193, 91)	0.73 (207, 77)
F-measure	0.49	0.49	0.73	0.64	**0.74**

Overall, for the prostate cancer dataset, our full approach achieves 39% improvement in precision (from 0.59 to 0.82) over the EMU approach with a sequence filter incorporated. Incorporation of a sequence filter improves the precision at the expense of recall for EMU, but for our approach, the addition of a sequence filter has the opposite effect–both precision and recall improve with the addition of the filter. This is because our approach evaluates each gene in the candidate list of genes and thus increases the likelihood that the final gene match is correct. Consequently, the overall F_1_-measure is 28% higher (from 0.62 to 0.794) than EMU’s F_1_-measure. Similarly for the breast cancer dataset, we find that the precision of the proposed approach is 22% higher (from 0.61 to 0.742) than the precision value for EMU, and the F_1_-measure is 15% higher (from 0.64 to 0.74). This is a significant improvement in the state of the art for disease-gene-variant triplet extraction. Moreover the balanced performance of our approach offers practical advantages for database curation: achieving high precision at the cost of a small decrease in recall suggests that the extracted results contain very few errors (false positives) with comparable coverage (recall).

### Extrinsic evaluation: Comparison with UniProtKB

To assess the potential of our approach in assisting database curation, we performed an extrinsic analysis by comparing our text-mined results against curated relationships for a total of ten diseases. UniProtKB–the Universal Protein Resource Knowledge Base–is a database of protein sequence and annotation data produced in Switzerland through collaboration between the European Bioinformatics Institute (EMBL-EBI), the Swiss Institute of Bioinformatics (SIB) and the Protein Information Resource (PIR) [38]. Its scope includes all human genes and function-altering gene variants along with any diseases caused by those variants [39]. Data collection from UniProtKB is explained in detail in the supplementary material, S4 Text. The raw output of our algorithm across the literature for these ten diseases can be found in the supplementary material (S1 Data) along with a thorough analysis of these results in S5 Text.

**Fig 4:**
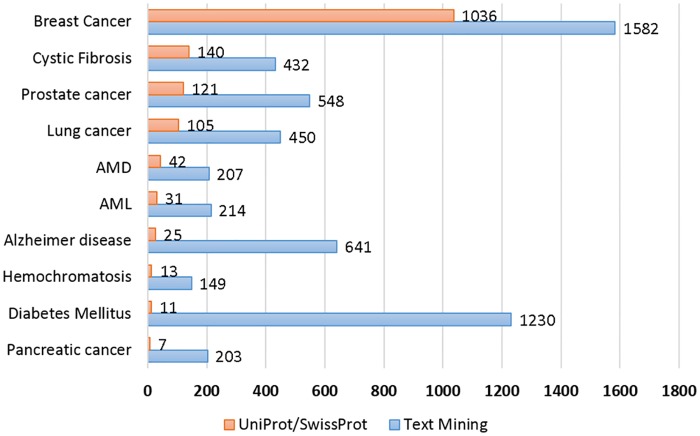
Comparing PubMed text-mined results with UniProtKB curated set.

We compare the text-mined results with the UniProt curated set. As shown in Fig 4, the red bars denote the counts of text-mined results for each disease, and the blue bars denote the counts of curated variants for each disease in the UniProtKB dataset. As is apparent in the figure, text mining extracts a significantly larger number of triplets than exist in UniProtKB curations.

**Fig 5:**
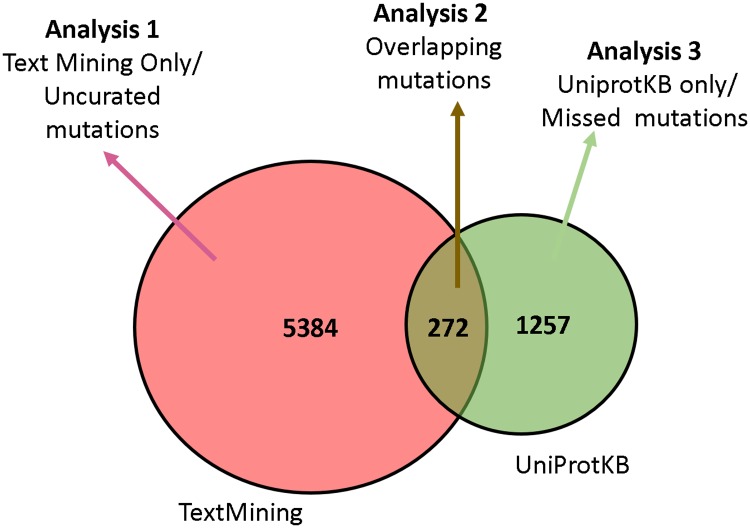
Three-tier analysis of text-mined vs. curated gene-disease-variant triplets.

From UniProtKB, we collected 1,529 unique gene-variant pairs for the ten diseases. In comparison, we extracted 5,656 gene-variant pairs from the literature using our text mining approach for the same diseases. We divided the UniProtKB entries and text-mined disease-gene-variant triplets into three separate groups by their overlap and evaluated the proposed approach differently in each group (shown in Fig 5). We evaluated the accuracy (in precision) of gene-variant pairs that were only found through text mining via human annotation of a stratified random sample of the results (Analysis 1). Overlapping mutations were evaluated directly with UniProtKB with respect to their gene association (Analysis 2). Finally, we analyzed gene-variant pairs that were unique to UniProtKB–potential false negatives for our approach (Analysis 3).

#### Analysis 1: Evaluation of un-curated mutations

Text mining returned 5,384 triplets that were not found in UniProtKB. These variants and their supporting literature references are potential candidates for curation. On the other hand, it is possible that these results contain false positives. We assessed the accuracy of this group via human judgment of a stratified random sample of the results for each disease (described below) given the absence of a gold standard.

In the random sampling, all 5,384 text-mined triplets were categorized into three representative groups based on their mutation frequency in literature: (a) 138 highly frequent mutations (seen in >10 PMIDs), (b) 343 moderately frequent mutations (4 to 10 PMIDs), and (c) 4903 low-frequency mutations (<4 PMIDs). We randomly sampled a maximum of 10, 15 and 30 instances from these high, moderate and low frequency mutation categories respectively. Repeating such sampling for ten diseases gave a total of 58, 112 and 260 instances (430 total) in the respective categories (Table 2). Thus this sample represents approximately 8% (430/5384) of the total uncurated mutations.

**Table 2:** Precision computation based on human annotation of random samples from uncurated mutations.

Frequency Group	High (138)	Medium (343)	Low (4903)	Total (5384)
**Correct triplets**	47	89	195	331
**Triplets evaluated**	58	112	260	430
**Accuracy (precision)**	0.81	0.80	0.75	0.77

In manually annotating these sampled mutations, it is not uncommon that–for any given gene-variant-disease triplet, separate publications disagree regarding whether or not a true association exists. For example, regarding the (NOS3 –Glu298Asp–Alzheimer disease) triplet, PMID 23952620 contains the following assertion:

…*no significant association was observed between the NOS3 Glu298Asp polymorphism and AD risk*.

However, PMID 16813604 contains the opposite assertion:

… *The meta-analysis showed a small effect of the Glu298Asp GG genotype on AD risk*.

Since we performed our comparison between our approach and UniProt at the triplet level and not at the level of the individual articles supporting those triplets, we adopted a simple set of rules for judging between disputing assertions: when disagreement between articles was identified, the association or lack of association was determined by the conclusions of review articles or meta-analyses if present. If no review or meta-analysis was available, the most recent articles dictated the final judgment. In the few instances when meta-analyses and equally recent articles supported opposite assertions, we permitted curators to use their own judgment after evaluating all the PMIDs. In this task, curators were permitted to refer to the full text of the articles for additional information.

The manual annotation of the sampled mutations thus proceeded in the following manner. A part (approximately 40%) of the sampled mutations for each disease was annotated by two human annotators independently. Each annotator performed two tasks, (i) labeling the text-mined mutation with its actual gene and disease association by reading the relevant literature; and (ii) marking ‘True’ when the text-mined triplet fully matched the actual gene and disease association (‘False’ otherwise). We evaluated the consistency of the manual annotators by comparing their respective sets for overlap and disagreements. We found an 81% inter-annotator agreement in their annotations. We review the insights that we gained from this exercise in the Discussion section. After that, one of the original annotators–a fourth-year medical student–proceeded to annotate the remaining 60% of the sampled mutations. Table 2 contains the results of these manual annotations.

As shown in Table 2, with regards to the disease-gene-variant triplets that were found only through text mining, the performance of our approach reaches a precision of 0.81 for highly frequent mutations and 0.80 and 0.75 for less frequent ones. For the aggregate of the mutations analyzed, the overall precision is 0.77. Each incorrect triplet can be classified by whether the error resulted from an incorrect gene, incorrect mutation, or incorrect disease. Likewise errors can be classified by whether they resulted from incorrect entity tagging (e.g. the entity “F442A” in PMID: 2960133 was incorrectly classified as a mutation when it is actually an adipocyte cell line) or from incorrect relationship extraction (e.g. in PMID: 21852217, I253M was incorrectly classified as relating to IRS1 when it is really a variant of IGFBP1). We formally evaluated all errors by frequency classification. Of a total 23% error rate, 8.4% came gene errors, 13.2% from disease-mutation misclassification errors and 1.4% from mutation errors. We provide a detailed breakdown of these errors in the supplementary material, S6 Text.

#### Analysis 2: Evaluation of overlapping mutations

In this category, a correct association between disease and variant has already been confirmed by the presence of this association in UniProtKB. Thus, the only remaining step to confirm the correctness of the full triplet is to assess whether the gene extracted via text mining matches the curated gene. Using the proposed approach, we were able to match gene-variant pairs with an average accuracy of 93% over all 10 diseases. Disease-wise analysis of the overlapping mutation is provided in the supplementary material, S1 Fig. Below we highlight the disease-wise analysis of the diseases where the accuracy falls below 95%.

From the gene comparison results, we find that the accuracy of extracted triplets in this group is over 95% for the majority of diseases with the exceptions of breast cancer (83.7%), cystic fibrosis (89.5%), Alzheimer’s disease (86.7%) and pancreatic cancer (66.7%). Several specific reasons account for the lower gene match for these four diseases:

Pancreatic cancer. We found there were only three overlapping mutations, thus a 66.7% match corresponds to a 2/3 match.

Breast cancer. Of the 14 mutations with a gene error, 11 of them were curated in UniProt from a single, large-scale study involving 13,023 genes for breast and colorectal cancer (PMID: 16959974). We discovered that neither the abstract nor the full text of this PMID contains any mention of the curated genes. Even after examining the supplementary material for this study we concluded it was not possible to definitively confirm a true gene association for these variants without additional sources of information.

Alzheimer’s disease (AD). In AD there were two gene match errors (out of 15 mutation overlaps). The first error came from an article (PMID: 10732806) with an abstract containing a mutation-gene pair–‘p.V148I’and ‘presenilin-2**’**–that our gene extraction tool missed. The second error occurred in PMID: 23415546 due to an error in gene identification for the mutation ‘p.A48V.’ The abstract for this PMID mentions two genes–PSEN1 and Cathepsin D (CSTD)–but GNormPlus identified only the PSEN1 gene and not CSTD, which is the actual gene associated with the mutation.

Cystic fibrosis (CF). The ten overlapping mutations with gene match errors were all in the CFTR gene. CFTR is the most prominent gene related to CF; however, the PMIDs referencing these mutations were not annotated with this gene mention; this was thus a gene extraction error.

#### Analysis 3: Evaluation of gene-variant-disease triplets present only in UniProtKB

A likely reason why these triplets were not returned by our text mining approach is that they are not mentioned in the abstract. Thus to verify this hypothesis, we obtained the reference PMIDs from UniProt for each triplet and used an automated approach to screen each abstract for the presence of the gene, mutation and disease information.

As explained in the Methods section, PubTator uses several state-of-the-art text mining techniques to annotate PubMed abstracts. Therefore we used PubTator to annotate the three entities—gene, mutation and disease—in the PubMed abstracts found in UniProt dataset but not in text mined results. These PubTator annotations were then compared with the triplets present only in the UniProtKB dataset. The comparison was performed at the concept level (i.e. each entity was normalized to a concept). The results of this analysis are shown in Table 3. PubTator tools annotate the entities with a reasonable accuracy for use in large-scale automatic extraction—87% (GnormPlus), 78% (DNorm) and 91% (tmVar). However, we also acknowledge that some errors may have occurred in this analysis as a result of using these automated tools.

**Table 3:** Analysis of missed mutations.

Disease	<Protein-Mutation-PMID >analyzed	Had disease mention	Had protein mention	Had mutation mention	Had triplet mention	#of PMIDs	Comments
**Breast cancer**	677	622	50	3	0	45	PMID: 16959974 had 587/677 mutations
**Cystic fibrosis**	19	5	4	7	0	15	
**Prostate cancer**	87	51	69	10	0	38	
**Lung cancer**	123	43	112	9	**3**	44	PMID: 17349580- classification error. PMID: 18227510- no lung cancer Mesh terms
**Age-related Macular Degeneration**	19	17	17	0	0	7	
**Acute Myeloid Leukemia**	49	29	45	6	**1**	26	PMID: 16247455: missed due to classification error
**Alzheimer’s disease**	15	8	12	0	0	10	
**Hemochromatosis**	1	0	1	1	0	1	
**Diabetes mellitus**	19	8	14	3	0	19	
**Pancreatic cancer**	5	1	4	0	0	5	

We found that breast cancer has the highest number of missed mutations, but a relatively low number of supporting PMIDs. As shown in the table, the PubTator analysis revealed that none of the 45 unique PMIDs had all the three entities within their abstract. Further manual analysis of these PMIDs showed that the large-scale study mentioned earlier (PMID: 16959974) accounted for 587 of the total missed mutations. PMID: 16959974 is a landmark paper entitled, “The consensus coding sequences of human breast and colorectal cancers.” It contains the results of an analysis of 13,023 genes. The curated mutations from results of this study largely came from data tables in the supplementary material. A similar analysis of the remaining diseases showed that none of the disease-related PMIDs except those for lung cancer (LCa) and AML contained full triplet matches of the missed mutations. For lung cancer and AML, these errors were due to two reasons: error of mutation-disease association (ML classifier error) and PMID missed in the initial PMID list for these diseases. As shown in Table 3, both these errors were relatively infrequent. It is possible that entities missed by PubTator in abstracts biased the results in this analysis so we also performed an ad hoc analysis of several of the PMIDs from this set to ensure that the PubTator tool was correctly identifying the entities, especially the sequence variants. Our ad hoc analysis confirmed the absence of mutation mentions in the abstracts. This was as expected because PubTator uses the tmVar tool to extract mutation mentions, and tmVar’s accuracy (F-measure) is over 90% for benchmark datasets for mutation identification [14]. Moreover, point substitutions, being the easiest variant form to identify with tmVar, will have a higher accuracy for discovery in the text. Hence, their absence in tmVar results most certainly imply that these mutation mentions were absent in the abstract.

In general, we infer from this analysis that our approach of text mining abstracts for mutations missed mutations most often because the complete information about the disease-mutation association was not present in the abstracts but rather was in the full text or supplementary data analysis of the supporting literature.

## Discussion

Based on the aforementioned three analyses of the text-mined mutations and the UniProt curated mutations, we state the following findings:

1. The uncurated triplets found by text mining are potentially good candidates for database curation. Moreover, text mining is able to find the related genes for these mutations with over 80% accuracy, which is consistent with the evaluation results on the two gold-standard benchmark datasets.
2. The performance validation of the text mining approach for the overlapping mutations shows that the text-mined results are comparable to human curation results.
3. Human curation results supersede the text-mined results when the mutations are not mentioned in the abstracts but are mentioned elsewhere in the full-text or supplementary data.

One reason that may explain the relatively low overlap between the results of our approach and the curations in UniProtKB is a difference in institutional focus. UniProtKB only curates gene variants that result in alterations of protein function, and while our approach does identify function-altering variants, it also includes a much broader range of associations, including disease-causing, protective, and treatment-response associations. Although we have presented our findings as a comparative analysis with data from the well-known UniProtKB, we nevertheless consider that the text mining results will act as a complimentary support to curators of any database to enhance the efficiency of the curation of disease mutations and genes. For instance, the uncurated text mined results may represent priority candidates for human curators during triage. A key step in making our approach useful for such databases will be developing additional text mining filters that can permit curators to retrieve custom sets of gene-variant-disease triplets with accompanying evidence that will best suit their institutional objectives.

An important consideration in the field of automated mining of gene-variant-disease associations from literature is that nomenclature standards for gene variants have evolved over time as researchers have understood new levels of genetic complexity [40–42]. One trend has been a movement to describe all variants by the sequence of the coding DNA strand and avoid other levels of description (i.e. mRNA and protein). As mentioned previously, our approach is fairly agnostic to variant nomenclature alterations because of the robust nature of tmVar’s mutation identification algorithm–we extract all types and descriptions of variants. Nevertheless, in this work, we concentrated on protein sequence nomenclature largely to facilitate comparison with the UniProtKB database. Since our algorithm incorporates a sequence filter and since protein sequences are notoriously variable, it is possible that this processing step may have removed correct variant associations with slightly different protein sequence numbering from our results. This could also explain some of the difference in overlap between our text-mined results and the curated associations in UniProtKB. Normalization of variant mentions in literature is an important next-step for automated extraction of genotype-phenotype relations.

**Discrepancies between human annotators**: As mentioned previously, two human annotators evaluated each text-mined result in approximately 40% of the sample set for each of the ten diseases. Following independent evaluation of each variant triplet, the annotators met and discussed the variants that they had rated differently and reached a consensus. A comparison of these specific instances revealed several trends. For example, disagreements were more common when sentence syntax was complex (e.g. PMID 22774841, disease: hemochromatosis, variant: W779X, gene: ATP7B –final judgment: no association), when the article in question addressed a disease related to but distinct from the disease in question (e.g. PMIDs 21853126, 21680267 and 18580449; disease: pancreatic cancer; variant: P86S, gene GCGR–final judgment: true association), when disagreement exists in the published literature about the significance of a given variant (e.g. PMID: 23397959, disease: AML, variant: K751Q, gene: ERCC2 (XPD) versus PMID: 24486506 for the same disease and variant–final judgment: true association), or when the genetic variants returned via text mining were the result of experimental modifications (e.g. PMID 18595696, disease: cystic fibrosis, variant: K1250A, gene: CFTR–final judgment: true association).

## Limitations and Future Directions

We identify a few areas of work that may enhance our approach and improve its utility for future research and other applications. First, although our approach robustly identifies gene variant mentions of different types across multiple nomenclature styles, we do not currently normalize variants. To avoid duplicate references in this work we have constrained our evaluation to only variant mentions at a protein level. Our work could be improved if we were to normalize all gene variant references to a single notation format, preferably to a complementary DNA sequence. Such normalization would facilitate future comparisons with data sources and also increase the utility of the sequence validation step in our approach. Still, this sequence validation step will only be possible for substitutions and deletions and not for insertion-type variants. This limitation did not affect our analysis in this work since UniProtKB only curates substitution variants and does not curate insertions or deletions. In future studies, we may need to incorporate a sequence validation method that will permit validation of insertions as well as substitutions and deletions.

Another important limitation of the current approach is that it mines information only from abstracts and not full text or supplementary material, which have been shown to be an important source of genetic variant information [43]. An extension to full text will require more advanced systems to overcome the additional noise in the full text and tables. As shown in the results of Analysis 3, we miss a large proportion of the UniProtKB mutations because they either appear in full text or supplementary material. For these reasons, an extension of the current work to full text is one of the important future steps of our efforts. Two potential resource for developing this extension are the Variome Corpus, which contains ten full-text articles with manual annotations applied according to the Variome Annotation Schema guidelines [44], and the Biomedical entity Relation ONtotlogy COrpus (BRONCO), which contains a large collection of annotated relationships between genes, variants, drugs, and cell lines from the full text of 108 articles [45]. Finally, this proposed framework directly uses several state-of-the-art tools (like tmVar, DNorm and GNormPlus). Future advances in the respective domains of these tools would enable increased performance of the proposed approach. This study has been helpful to excavate several examples which will serve as feedback to the independent machineries of mutation, gene, and disease annotation systems.

In conclusion, we have shown that our approach for text mining disease mutations and their associated genes from the biomedical literature is successful. We have also shown that the training step for this approach is generalizable among different types of diseases. Our approach can thus apply broadly to a variety of diseases.

The intrinsic evaluation shows that our approach achieves state-of-the-art performance and compares favorably to a competitive system. Our comparative analysis with real-world curation data confirms the accuracy of our approach and demonstrates that text-mined results may be potentially useful for expanding the coverage of curation and improving curation quality.

## Supporting Information

S1 Text Machine learning algorithm for mining disease-mutation relationships. (DOCX)

S2 Text Results for other types of mutations identified using text mining. (DOCX)

S3 Text Performance without Bing. (DOCX)

S4 Text Data collection from UniProtKB. (DOCX)

S5 Text Disease-wise analysis statistics. (DOCX)

S6 Text Error analysis. (DOCX)

S1 Fig Disease-wise analysis of disease-gene variant accuracy. (TIF)

S1 Data File with raw results. (TXT)
