# Supplementary material for: Text Mining Genotype-Phenotype Relationships from Biomedical Literature for Database Curation and Precision Medicine
Source: PLoS Comput Biol. 2016 Nov 30;12(11):e1005017. doi: 10.1371/journal.pcbi.1005017 (PMC5130168; doi:10.1371/journal.pcbi.1005017)
Supplement: S1 Text — (DOCX) [file pcbi.1005017.s001.docx]

**S1 Text. Machine learning algorithm for mining disease-mutation relationships:**

We briefly discuss the machine learning and text mining approach we developed to extract disease-mutation relationships from PubMed abstracts. As shown in Supplementary Figure 1 (below), using the tmVar and DNorm entity recognition tools for mutation and disease mentions respectively, we determine an association (yes or no) by computing six quantitative features from the text in the abstract. These 6 features are constructed to capture various information types such as statistical information about frequency, inter-entity distances, and text sentiment and its strength. We use a pre-trained (on a human annotated benchmark dataset) machine learning model to make decisions about associations using the six feature set.


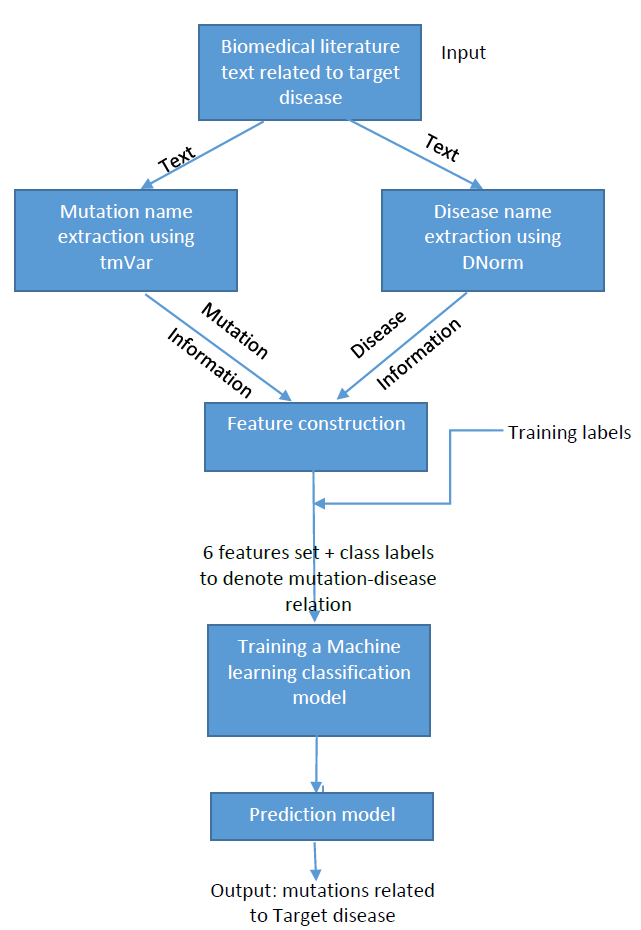


Supplementary Figure 1: Flowchart of our disease-mutation association mining algorithm.
