# Supplementary material for: Text Mining Genotype-Phenotype Relationships from Biomedical Literature for Database Curation and Precision Medicine
Source: PLoS Comput Biol. 2016 Nov 30;12(11):e1005017. doi: 10.1371/journal.pcbi.1005017 (PMC5130168; doi:10.1371/journal.pcbi.1005017)
Supplement: S2 Text — (DOCX) [file pcbi.1005017.s002.docx]

**S2 Text. Results for other types of mutations identified using text mining:**

In Supplementary Figure 2 (below) we show a comparative view of all the different types of mutations text mined from PubMed abstract for the 10 diseases. As shown in the figure, except for the mutations mentioned using their rs# number, the mutation of deletions, insertions, Frame Shift types are significantly lower in number than the substitutions. Due to lack of any gold standard dataset for such categories, we have not evaluated the accuracy of triplets that involve these types mutations. Since the gene information for most SNPs is accurately curated in the dbSNP database, we directly used the results from dbSNP using the e-utility tool of NCBI. For those mutations without entries in dbSNP, we used a text mining approach. However, we did not undertake any accuracy evaluation for these mutations.


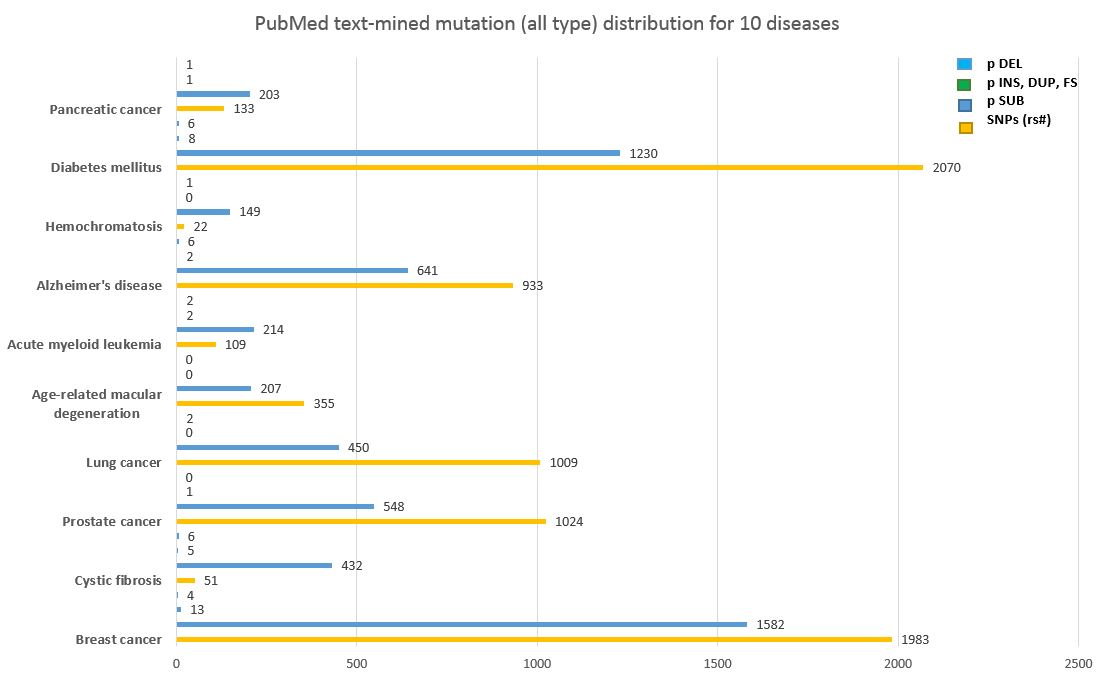


Supplementary Figure 2. Frequency distribution of different mutation types for 10 diseases.
