# Supplementary material for: Text Mining Genotype-Phenotype Relationships from Biomedical Literature for Database Curation and Precision Medicine
Source: PLoS Comput Biol. 2016 Nov 30;12(11):e1005017. doi: 10.1371/journal.pcbi.1005017 (PMC5130168; doi:10.1371/journal.pcbi.1005017)
Supplement: S3 Text — (DOCX) [file pcbi.1005017.s003.docx]

**S3 Text. Performance without Bing:**

In this experiment, we use randomly drawn samples of text mined results for 10 diseases to infer the impact of Bing ranking on the performance of our system. Although this experiment can also be done with the EMU datasets for PCA and BCA, the restricted size of PMIDs in those datasets limits the performance of our PubMed-based ranking approach and thus the overall performance. We, therefore, choose the humanly annotated randomly sampled dataset for 10 diseases to compare the performance of the approach with and without using Bing ranking in addition to PubMed ranking.

As shown in Supplementary Table 3, removing Bing ranking for gene identification, lowers the precision by 6% as compared with a system that uses Bing ranking in combination with PubMed ranking.

|  | High Freq | Med Freq | Low freq | Total | Precision |
| --- | --- | --- | --- | --- | --- |
| Total | 58 | 112 | 260 | 430 |  |
| Correctly predicted with Bing | 47 (81%) | 89 (80%) | 195 (75%) | 331 | 77% |
| Correctly predicted without Bing | 43 (74%) | 83 (74%) | 181(70%) | 307 | 71% |

Supplementary Table 3: Results of removing Bing for 10 diseases using randomly evaluated samples.
