# Supplementary material for: Text Mining Genotype-Phenotype Relationships from Biomedical Literature for Database Curation and Precision Medicine
Source: PLoS Comput Biol. 2016 Nov 30;12(11):e1005017. doi: 10.1371/journal.pcbi.1005017 (PMC5130168; doi:10.1371/journal.pcbi.1005017)
Supplement: S4 Text — (DOCX) [file pcbi.1005017.s004.docx]

**S4 Text. Data collection from UniProtKB**

### The procedure for our data collection from UniProtKB is explained below.

### We collected the latest version of the UniProt database by downloading the search results for the query: *homo sapiens AND reviewed:yes AND organism:"Homo sapiens (Human) [9606]"*.  There were a total of 20,196 protein hits for this query on October 10, 2015. These data were collected in a text file that was later processed to reflect information about the curated protein-mutations (substitutions only) for each of the ten diseases. The processing involved the following steps:

1. Extract protein IDs from the line with the header 'AC'.
2. Extract gene names from the line with the header 'GN'**.**
3. Extract variants from the line with the header 'FT' and the sub-header 'VARIANT'. This information contains the mutation position as well as the identities of the wild type and mutant amino acids.
4. Extract, from the curator notes in the variant line, the disease names associated with all proteins for each variant/mutation. Since concept-level disease references in the human curation for the variant were absent, identifying the associated disease mentions was challenging. We, therefore, used a mapping (shown in Supplementary Table 1) to identify disease mentions in the curator's notes.
5. Extract PMID references for each curated variant. If this information was available, it was present in the curator's notes for the variant.

Supplementary Table 1: Mapping for searching disease mention in curator's notes

| **Actual disease** | **Synonyms searched in the text** |
| --- | --- |
| Breast cancer | BC, breast cancer, breast carcinoma |
| Cystic fibrosis | CF, cystic fibrosis |
| Prostate cancer | PC, prostate cancer, prostate carcinoma |
| Lung cancer | NSCLC, lung cancer, lung carcinoma, nonsmall cell lung cancer, alveolar cell carcinoma |
| Age-related macular degeneration | ARMD, macular degeneration |
| Acute myeloid leukemia | AML, acute myeloid leukemia |
| Alzheimer's disease | AD, AD1, alzheimer's disease, Alzheimer disease |
| Hemochromatosis | HFE,HFE1, hemochromatosis |
| Diabetes mellitus | IDDM,IDDM1, diabetes mellitus |
| Pancreatic cancer | Pancreatic cancer, pancreatic carcinoma |
