# Supplementary material for: Text Mining Genotype-Phenotype Relationships from Biomedical Literature for Database Curation and Precision Medicine
Source: PLoS Comput Biol. 2016 Nov 30;12(11):e1005017. doi: 10.1371/journal.pcbi.1005017 (PMC5130168; doi:10.1371/journal.pcbi.1005017)
Supplement: S5 Text — (DOCX) [file pcbi.1005017.s005.docx]

**S5 Text. Disease-wise analysis statistics:**

Supplementary Table 2 shows the statistics describing our disease-wise analysis of PMIDs containing mutation, gene and disease mentions and pairwise occurrences.

| Disease Name | Total PMIDs | Had Disease Mention | Had Gene mention | Had Mutation Mention | Had Gene-Mutation mention | Had Gene-Disease mention | Had Disease-Mutation mention | Had Disease-Gene-Mutation mention |
| --- | --- | --- | --- | --- | --- | --- | --- | --- |
| Acute myeloid leukemia | 29786 | 28130 | 15415 | 513 | 453 | 14482 | 489 | 432 |
| Cystic fibrosis | 18326 | 17985 | 4039 | 1026 | 270 | 3963 | 1017 | 265 |
| Diabetes mellitus | 202181 | 198928 | 94332 | 3929 | 3648 | 93260 | 3906 | 3631 |
| Hemochromatosis | 3234 | 3154 | 1822 | 1045 | 904 | 1792 | 1038 | 900 |
| LungCancer | 79164 | 78826 | 33981 | 2633 | 2321 | 33886 | 2627 | 2317 |
| Pancreatic cancer | 37290 | 36422 | 16757 | 579 | 515 | 16360 | 571 | 510 |
| Alzheimer’s | 74978 | 61790 | 29560 | 2786 | 2437 | 28329 | 2730 | 2395 |
| Age-related macular degeneration | 11384 | 11331 | 4389 | 803 | 747 | 4377 | 803 | 747 |
| Breast cancer | 155512 | 154815 | 78852 | 6526 | 5866 | 78580 | 6501 | 5843 |
| Prostate cancer | 66320 | 66031 | 39637 | 2011 | 1760 | 39524 | 2003 | 1752 |
| Total | 678175 | 657412 | 318784 | 21851 | 18921 | 314553 | 21685 | 18792 |
| Percentage | | 96.94 | 47.01 | 3.22 | 2.79 | 46.38 | 3.20 | 2.77 |

Supplementary Table 2 – Disease-wise analysis statistics
