# Supplementary material for: Text Mining Genotype-Phenotype Relationships from Biomedical Literature for Database Curation and Precision Medicine
Source: PLoS Comput Biol. 2016 Nov 30;12(11):e1005017. doi: 10.1371/journal.pcbi.1005017 (PMC5130168; doi:10.1371/journal.pcbi.1005017)
Supplement: S6 Text — (DOCX) [file pcbi.1005017.s006.docx]

**S6 Text. : Error analysis**

In this supplementary section we present an analysis of the incorrect triplets identified in our validation step. Each incorrect triplet can be classified by whether the error resulted from an incorrect gene, incorrect mutation, or incorrect disease. We investigated the proportion of error by the frequency of mentions of that triplet, and we present the results of our analysis in Supplementary Figure 3. As described in the main article, high frequency refers to triplets with >10 mentions, low refers to triplets with <4 mentions, and medium frequency mentions have 4-10 mentions.

For gene and disease error, it can been seen that error counts increase as the frequency of triplets decreases. Analysis of the error in assigning genes to disease-variant pairs reveals that the high error rate in low-frequency mutations is most likely due to a lack of global context to assign a gene to a mutation. By definition, there is limited or absent global context for low frequency gene-mutation mentions, hence the gene assignment for low frequency mutations is done using local context information only. As a result, multiple gene occurrences in the text more readily confound the results and contribute to this error. Still it is surprising that several gene errors occurred in the high frequency category where one would expect enough PMIDs to support the extracted gene. After investigating these errors, we found that as the number of PMID references increases, the number of candidate genes that have to be ranked also increases. In these cases the lists of candidate genes often include several common gene names that appear frequently in the literature for a particular disease e.g. BRCA1 for breast cancer or CFH for AMD. It is possible for these common genes to outnumber the actual genes both in the literature as well as in Bing search results.

We formally evaluated all errors by frequency classification. Of a total 23% (99 of 430) error rate, 8.4% (36 of 430) came from gene errors, 13.2% (57 of 430) from disease-mutation misclassification errors and 1.4% (6 of 430) from mutation errors caused by false mutation mention identification by the tmVar algorithm. To explore the disease errors, we analyzed the three frequency categories and found the same trend seen with gene errors of increasing error with decreasing frequency. For these errors, wrong disease association occurred most frequently as a consequence of classification error of our machine learning algorithm.


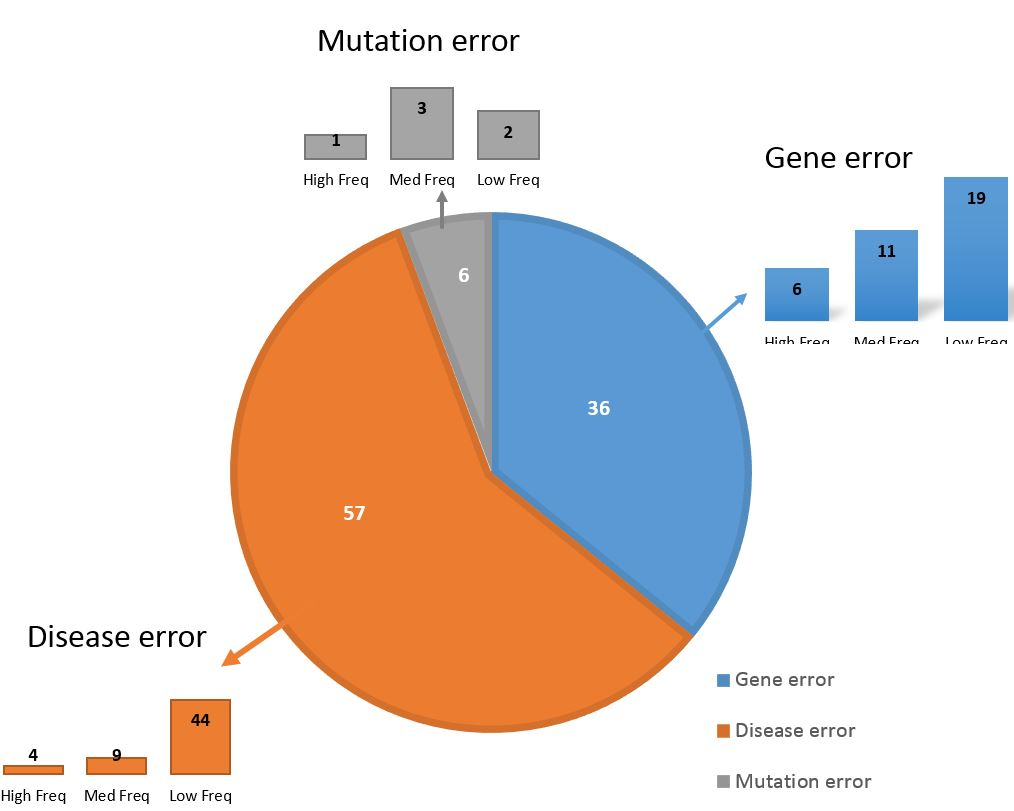


Supplementary Figure 3. A pie chart analysis of errors in triplet extraction results.
