# Supplementary figures and images for: Text Mining Genotype-Phenotype Relationships from Biomedical Literature for Database Curation and Precision Medicine
Source: PLoS Comput Biol. 2016 Nov 30;12(11):e1005017. doi: 10.1371/journal.pcbi.1005017 (PMC5130168; doi:10.1371/journal.pcbi.1005017)

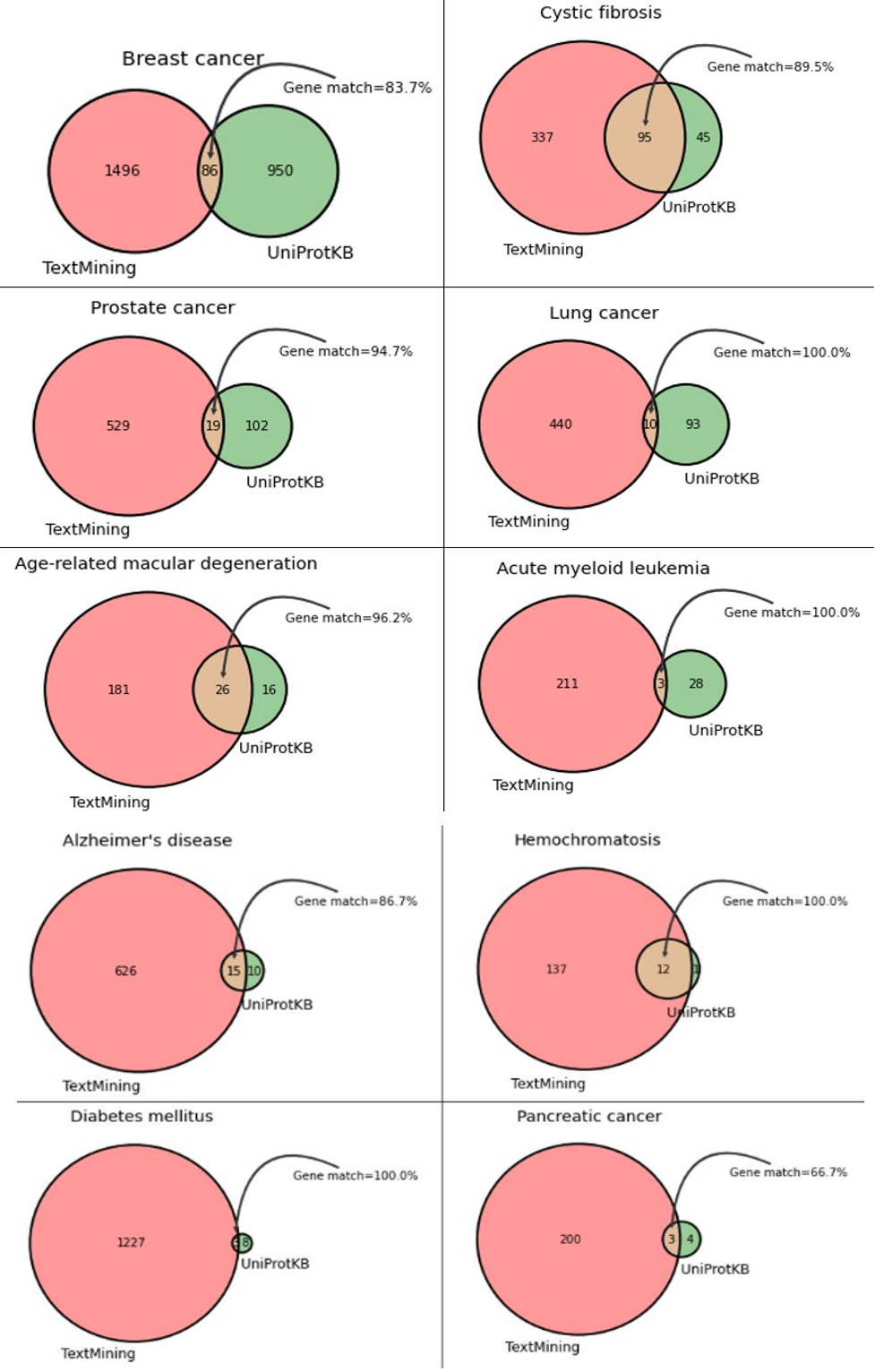

Supplement: S1 Fig — (TIF) [file pcbi.1005017.s007.tif]
